# Do miRNAs Play a Role in Fetal Growth Restriction? A Fresh Look to a Busy Corner

**DOI:** 10.1155/2017/6073167

**Published:** 2017-03-29

**Authors:** Benito Chiofalo, Antonio Simone Laganà, Alberto Vaiarelli, Valentina Lucia La Rosa, Diego Rossetti, Vittorio Palmara, Gaetano Valenti, Agnese Maria Chiara Rapisarda, Roberta Granese, Fabrizio Sapia, Onofrio Triolo, Salvatore Giovanni Vitale

**Affiliations:** ^1^Unit of Gynecology and Obstetrics, Department of Human Pathology in Adulthood and Childhood “G. Barresi”, University of Messina, Messina, Italy; ^2^Unit of Psychodiagnostics and Clinical Psychology, University of Catania, Catania, Italy; ^3^Department of Maternal and Child Health, Gavardo Hospital, Brescia, Italy; ^4^Department of General Surgery and Medical Surgical Specialties, University of Catania, Catania, Italy

## Abstract

Placenta is the crucial organ for embryo and fetus development and plays a critical role in the development of fetal growth restriction (FGR). There are increasing evidences on the role of microRNAs (miRNAs) in a variety of pregnancy-related complications such as preeclampsia and FGR. More than 1880 miRNAs have been reported in humans and most of them are expressed in placenta. In this paper, we aimed to review the current evidence about the topic. According to retrieved data, controversial results about placental expression of miRNAs could be due (at least in part) to the different experimental methods used by different groups. Despite the fact that several authors have demonstrated a relatively easy and feasible detection of some miRNAs in maternal whole peripheral blood, costs of these tests should be reduced in order to increase cohorts and have stronger evidence. In this regard, we take the opportunity to solicit future studies on large cohort and adequate statistical power, in order to identify a panel of biomarkers on maternal peripheral blood for early diagnosis of FGR.

## 1. Introduction

Fetal growth restriction (FGR) refers to a condition in which there is the stop or the decrease of the genetic determined potential growth of a fetus during pregnancy. FGR is due to different causes, including maternal smoking, undernutrition, infection, and congenital abnormalities; conversely, if it is not possible to individuate a clear cause, it is defined as idiopathic. FGR is often associated with preeclampsia [1] and represents the most common pregnancy complication, accounting for about a third of all preterm births. Usually FGR is diagnosed when, through ultrasound, fetal weight is estimated to be less than the 10th percentile for gestational age using a validated fetal growth scale [2]. The most studied cause of FGR in animal models is maternal calorie restriction during gestation, but while much is known about the consequences of this deprivation, molecular mechanisms that underlie these conditions still remain unclear.

Placenta is the crucial organ for embryo and fetus development and plays a critical role in the development of FGR. In this regard, FGR could be considered as a placentation disorder, derived from a dysregulation in trophoblast invasion with characteristic tissue morphology that leads to uteroplacental insufficiency. This condition would greatly benefit from the availability of early diagnostic tests to give an opportunity for early intervention or prevention, to improve maternal-fetal outcomes, and to substantially contain the public health costs.

There are increasing evidences on the role of microRNAs (miRNAs) in a variety of pregnancy-related complications such as preeclampsia and fetal growth restriction. More than 1880 miRNAs have been reported in humans and most of them are expressed in placenta. This kind of nucleic acid belongs to the family of small noncoding RNAs of on average 22 nucleotides in length, which regulates gene expression at the posttranscriptional level, inhibiting translation or promoting specific mRNAs degradation through interaction with the 3′ untranslated region [3, 4]. In detail, miRNAs seem to modulate cell development, differentiation, and proliferation, cell type-specific function, and are involved in the pathogenesis of many human diseases [5]. In several cases, miRNA expression is tissue-specific and, in addition, is significantly different between physiology and pathological conditions: for these reasons, investigations about miRNAs gained increasing attention for the possibility of future application in clinical diagnostics [6, 7].

Starting from these considerations, we aimed to review the current literature focusing on the role of miRNAs in FGR.

## 2. Materials and Methods

We performed a selective literature search of articles in English language, published from 2002 to 2017 and indexed in PubMed. We searched the following Medical Subject Headings (MeSH): “MicroRNAs” AND “Fetal Growth Retardation”. The initial database screening was performed by three authors (Laganà AS, Vaiarelli A, La Rosa VL), who were blinded to the aim of the study. Subsequently, other three authors (Chiofalo B, Rossetti D, Vitale SG) selected relevant information from the screened literature. We considered eligible all original manuscripts (randomized, observational, and retrospective studies), case series, and case reports. Furthermore, we extracted relevant information from selected reviews.

## 3. Results

### 3.1. Placental MicroRNAs

Several studies focused their attention on the expression of different miRNAs in placentas using real-time-PCR. Cindrova-Davies et al. [8] analyzed miRNA-21 expression from placentas of a small cohort (*n* = 6) of early-onset FGR cases and found its significant upregulation. Guo et al. [9] identified a significant downregulation of miRNA-194 in placentas from 26 FGR cases and from 16 preeclamptic women (16), compared to those from 29 normal pregnancies (29). Hromadnikova et al. [10] for the first time explored, in two different experiments, the placental expression profile of miRNAs known to be involved in cardiovascular and cerebrovascular diseases. They found that upregulation of miR-499a-5p is a common feature of all placental insufficiencies such as preeclampsia (*n* = 80), gestational hypertension (*n* = 35), and FGR (*n* = 35); in addition, they demonstrated an upregulation of miR-1-3p in FGR pregnancies with abnormal umbilical fetal flows (*n* = 19); finally, they found downregulation of a series of miRNAs (miR-16-5p, miR-26a-5p, miR-100-5p, miR-103a-3p, miR-122-5p, miR-125b-5p, miR-126-3p, miR-143-3p, miR-145-5p, miR-195-5p, miR-199a-5p, miR-221-3p, miR-342-3p, and miR-574-3p) in FGR requiring the delivery before 34 weeks of gestation.

Other authors studied miRNA-424 and its target gene (mitogen-activated protein kinase) that play a role in endothelial cell proliferation through fibroblast growth factor receptor 1 and regulate vascular endothelial growth factor [11]. According to their data analysis, the levels of this miRNA are increased in placentas from 25 FGR pregnancies compared with 25 placentas from uncomplicated pregnancies, suggesting that miRNA-424 is involved in placental disorders. Another study by Su et al. [12] searched, in a cohort of placentas, the miRNAs that regulate endocrine gland derived vascular endothelial growth factor (EG-VEGF) expression: they concluded that miR-346 and miR-582-3p regulate EG-VEGF-induced trophoblast invasion through repressing metalloproteinases 2 and 9. In addition, FGR placental tissues show an aberrant high expression level of miR-141, suggesting that this miRNA might play important roles in the pathogenesis of the disease by suppressing E2F transcription factor 3 and pleomorphic adenoma gene 1 [13].

To date, many studies focused their attention on chromosome 19 miRNA cluster (C19MC) [14–16]. In detail, C19MC comprises 46 miRNAs and is the largest gene cluster of miRNAs in humans, exclusively expressed in undifferentiated cells and in placenta. In this regard, comparing 14 placentas from FGR pregnancies with 14 from normal pregnancies, it was recently found that hypoxic stress does not affect C19MC miRNA expression, except for downregulation of miR-500c-3p [14]. Similarly, Hromadnikova et al. [17] detected a downregulation of 6 miRNAs (miR-517-5p, miR-518f-5p, miR-519a, miR-519d, miR-520a-5p, and miR-525) in placental tissues of 36 FGR pregnancies: compared to the previous studies, these results seem more robust since that authors investigated more types of miRNAs and used those that were previously demonstrated to be exclusively expressed or highly expressed in placental tissues. The significantly decreased expression of miR-519d, but not of miR-520a-5p and miR-525, was also confirmed by others on a larger cohort (50 healthy pregnancies compared with 45 FGR cases) [15]. Nevertheless, other experiments found that the expression of miR-518b was decreased, whereas miR-519a was significantly increased, in 30 FGR placentas [16]. Some of these miRNAs studied in human placentae were also studied in animal models.

### 3.2. Circulating miRNAs

During pregnancy, due to a normal extravillous trophoblast invasion, nucleic acids of the placental compartment are released into the maternal circulation: this release occurs through the migration of microvesicles from apoptotic/necrotic cells and active cellular communication system, involving also nanovesicles/exosomes and subcellular fragments [18, 19]. Due to placental continuous remodeling, these extracellular nucleic acids may be detected in maternal blood during the course of gestation and can be measured to monitor placental function and allow early diagnosis of pregnancy complications [20–23]. For these motivations in recent years there has been a trend to develop noninvasive methods for the detection in maternal circulation of cell-free nucleic acids, including miRNAs coming from the embryo-placental compartment [24–43]. Some studies detected FGR-specific miRNA expression changes in placentas, but these differences were not detectable in plasma [15–44]. A significant elevation of several extracellular placenta-specific miRNA levels was recently showed (miR-516-5p, miR-517, miR-518b, miR-520a, miR-520h, miR-525, and miR-526a,) during early gestation in 7 pregnancies with later onset of preeclampsia and/or FGR [44]. According to these data, an early screening (i.e., within the 12th to 16th weeks) of miRNA circulating levels may differentiate between women with normally progressing pregnancies and those who could later develop placental insufficiency–related complications [44]. Nevertheless, recent data showed that C19MC microRNAs might play a role in the pathogenesis of preeclampsia, but not of FGR [45]. Last year, Hromadnikova's group investigated maternal blood levels of specific miRNAs involved in cardiovascular and cerebrovascular diseases, finding a downregulation of miR-100-5p, miR-125b-5p, and miR-199a-5p in 39 patients with gestational hypertension, in 68 with preeclampsia, and in 33 with fetal growth restriction compared with 55 healthy controls; in addition, they showed downregulation of miR-17-5p, miR-146a-5p, miR-221-3p, and miR-574-3p only in FGR pregnancies [46]. In a small-scale analysis, others found that a group of miRNAs that are altered by hypoxia in trophoblasts (miR-27a, miR-30d, miR-141, miR-200c, miR-424, miR-205 and miR-451, miR-491, miR-517a, miR-518b, miR-518e, and miR-524) is elevated in FGR pregnancies (*n* = 14 FGR versus *n* = 14 controls) [47].

Some of these miRNAs, such as miR-141, miR-200c, and miR-205, were studied also in animal models [48, 49]. In particular, it was found that they play important roles in the maintenance of the integrity of the folded trophoblast-endometrial epithelial bilayer in porcine placentas [48].

## 4. Discussion

Based on the abovementioned data, miRNAs seem to be involved in placental development and consequently in placenta related disorders. As showed in Table 1, controversial results among these studies in placental expression of miRNAs could be due, at least in part, to the different experimental methods used by different groups. Despite the fact that several authors have demonstrated a relatively easy and feasible detection of some miRNAs in maternal whole peripheral blood [44–47], costs of these tests should be reduced in order to increase cohorts and have stronger evidence.

In this regard, we acknowledge that it may be extremely important to address future research directions taking into account the already available data from in vitro experiments and animal models: indeed, accumulating evidence suggests that miR141-3p and miR-200a-3p play a pivotal role for placental development in mouse and regulate the expression of insulin-like growth factor 2 [50]. Interestingly, upregulation of miR-125b was found to reduce significantly ethanol-induced caspase-3 activation and to diminish ethanol-induced growth retardation in mouse embryos [51], suggesting a possible protective role that is worthy of further investigation. Conversely, miR-24 and miR-103-2, which are related to adipocyte development, were both increased in low birth weight male guinea pig pups [52]. Probably this last element could be further confirmed in future studies, since several sex-specific effects were already found to be more pronounced in males with respect to females [53]. Last but not least, recent data showed that FGR is associated with increased lung miR-126-3p levels, which is known to modulate the expression of angiogenic factor, in rats [54]. The importance of angiogenic regulatory pathways was also highlighted by the abnormal upregulated expression of miR-127, miR-21, and miR-16 in placentas of deceased cloned sheep with respect to controls [55]. These data are extremely fascinating, since miR-21 expression was associated with increased vascular resistance also in growth-restricted human pregnancies [8, 56].

As suggested by accumulating evidence, miRNAs play also a pivotal role in epigenetic processes [57, 58]. Epigenetic mechanisms include DNA methylation, imprinting, and RNA transcriptional regulation through RNA molecules, such as miRNAs. These processes are influenced by multiple factors: intrauterine nutrient availability (determined by maternal nutrition and placental function) [59–62], maternal age [63, 64], use of drugs [65, 66], endocrine disruptors [67], toxins, and infectious agents. For this reason, integrated assessment of early pregnancy should evaluate a combination of biomarkers and ultrasound [68–73]. In addition, we take the opportunity to stress how future investigations about miRNA levels in both sera and placentas should evaluate the possible overlapping among preeclampsia, FGR, and gestational diabetes, since they all have in common placental vascular alterations due to angiogenic disbalance [74].

It is however clear that epigenetic information is transmitted, and potentially inherited, across generations through the remodeling of chromatin states. In this regard, selective miRNA expression may be involved in FGR through epigenetic mechanism.

## 5. Conclusion

Understanding which miRNAs are associated with the onset/progression of FGR seems mandatory to improve early diagnosis and management of the disease. In this regard, we take the opportunity to solicit future studies on large cohort and adequate statistical power, in order to identify a panel of biomarkers on maternal peripheral blood for early diagnosis of FGR.

## Figures and Tables

**Table 1:** Summary of relevant information from selected studies.

Authors, year	Type of study	Enrolled population	Methods of analysis	Samples	Main findings
Cindrova-Davies et al., 2013 [8]	Case-control	6 FGR, 6 PE-AD, 7 PE-ND, 7 HP (controls)	RT-qPCR	Placentas	Expression of miR-21 was significantly upregulated in PE-AD and FGR cases, but not in PE-ND cases

Guo et al., 2013 [9]	Case-control	26 FGR, 16 PE, 29 HP (controls)	RT-qPCR	Placentas	Downregulation of miR-149 was shown in PE cases; miR-194 was significantly downregulated in FGR and PE cases

Hromadnikova et al., 2015 [10]	Case-control	35 GH, 80 PE, 35 FGR, 20 HP (controls)	RT-qPCR	Placentas	(i) Upregulation of miR-499a-5p in PE, GH, FGR cases; (ii) upregulation of miR-1-3p FGR-AD in PE cases delivering after 34 weeks; (iii) downregulation of miR-26a-5p, miR-103a-3p, miR-145-5p in PE and FGR cases requiring delivery before 34 weeks; (iv) downregulation of miR-16-5p, miR-100-5p, miR-122-5p, miR-125b-5p, miR-126-3p, miR-143-3p, miR-195-5p, miR-199a-5p, miR-221-3p, miR-342-3p, miR-574-3p in FGR cases requiring delivery before 34 weeks

Huang et al., 2013 [11]	Case-control	25 FGR, 25 HP (controls)	RT-qPCR	Placentas	Upregulation of miR-424

Tang et al., 2013 [13]	Case-control	21 FGR, 34 HP (controls)	RT-qPCR	Placentas	High expression level of miR-141 in FGR cases

Donker et al., 2012 [14]	Case-control	14 FGR, 14 HP (controls)	RT-qPCR	Placentas	Hypoxic stress does not affect C19MC miRNA expression, except for downregulation of miR-500c-3p

Higasijima et al., 2013 [15]	Two-step case-control	First step: 45 FGR, 50 HP (controls)	RT-qPCR	Placentas	(i) miR-518b, miR-1323, miR-516b, miR-515-5p, miR-520h, miR-519d, miR-526b were significantly lower in FGR placentas than in controls; (ii) no differences in miR-516a-5p, miR-525-5p, miR-520a-5p levels
		Second step: 10 FGR, 10 HP (controls)	RT-qPCR	Maternal plasma/serum	No differences were found in miR-518b, hsa-miR-1323, hsa-miR-520h, hsa-miR-519d levels between FGR cases and controls

Wang et al., 2014 [16]	Case-control	30 FGR, 30 LGA, 30 NGA (controls)	RT-qPCR	Placentas	Decreased expression of miR-518b and increased expression of miR-519a in FGR cases with respect to NGA and LGA cases

Hromadnikova et al., 2015 [17]	Case-control	21 GH, 63 PE, 36 FGR, 42 HP (controls)	RT-qPCR	Placentas	(i) Downregulation of miR-517-5p, miR-519d, miR-520a-5p, miR-525 in GH cases; (ii) downregulation of miR-517-5p, miR-518f-5p, miR-519a, miR-519d, miR-520a-5p, miR-525 in FGR cases; (iii) downregulation of miR-515-5p, miR-517-5p, miR-518b, miR-518f-5p, miR-519a, miR-519d, miR-520a-5p, miR-520h, miR-524-5p, miR-525, miR-526a in PE cases

Hromadnikova et al., 2012 [44]	Case-control	16 PE, 11 FGR, 5 PE and FGR, 7 normal pregnancies with later onset of PE and/or FGR, 10 NP (controls), 50 HP (controls)	RT-qPCR	Maternal plasma/serum	(i) No differences were found in miR-516-5p, miR-517, miR-518b, miR-520a, miR-520h, miR-525, miR-526a concentrations between pathologic and normal pregnancies; (ii) significant elevation of miR-516-5p, miR-517, miR-518b, miR-520a, miR-520h, miR-525, miR-526a levels during early gestation in pregnancies with later onset of PE and/or FGR

Hromadnikova et al., 2013 [45]	Case-control	63 PE, 27 FGR, 23 GH, 55 HP (controls)	RT-qPCR	Maternal plasma/serum	(i) Plasmatic levels of miR-516-5p, miR-517, miR-520a, miR-525, miR-526a did not differ between FGR, GH, and HP patients; (ii) increased plasmatic levels and high expression of miR-516-5p, miR-517, miR-520a, miR-525, miR-526a were found in PE patients

Hromadnikova et al., 2016 [46]	Case-control	39 GH, 68 PE, 33 FGR, 20 HP (controls)	RT-qPCR	Maternal plasma/serum	(i) Downregulation of miR-100-5p, miR-125b-5p, miR-199a-5p in GH, PE, FGR cases compared with controls; (ii) downregulation of miR-17-5p, miR-146a-5p, miR-221-3p, miR-574-3p in FGR cases; (iii) downregulation of miR-100-5p, miR-125b-5p in PE cases; (iv) downregulation of miR-100-5p, miR-125b-5p, miR-146a-5p, miR-199a-5p, miR-221-3p, miR-574-3p in FGR cases; (v) downregulation of miR-100-5p, miR-125b-5p, miR-199a-5p in patients with GH

Mouillet et al., 2010 [47]	Case-control	14 FGR, 14 HP (controls)	RT-qPCR	Maternal plasma/serum	(i) No differences were found in the level of individual miRNA (miR-27a, miR-30d, miR-141, miR-200c, miR-424, miR-205 and miR-451, miR-491, miR-517a, miR-518b, miR-518e, miR-524) in FGR and HP cases; (ii) When considered as a group, the level of all tested miRNA species was elevated in plasma samples from women with FGR compared to controls

HP: healthy pregnancies; FGR: fetal growth restriction; PE: preeclampsia; -AD: abnormal fetal-Doppler; -ND: normal fetal-Doppler; GH: gestational hypertension; LGA: large for gestational age; NGA: normal for gestational age; NP: nonpregnant; RT-qPCR: real-time quantitative reverse transcription polymerase chain reaction.
